# Root Transcriptomic Analysis Reveals Global Changes Induced by Systemic Infection of *Solanum lycopersicum* with Mild and Severe Variants of Potato Spindle Tuber Viroid

**DOI:** 10.3390/v11110992

**Published:** 2019-10-29

**Authors:** Anna Góra-Sochacka, Aneta Więsyk, Anna Fogtman, Maciej Lirski, Włodzimierz Zagórski-Ostoja

**Affiliations:** Institute of Biochemistry and Biophysics Polish Academy of Sciences, Pawińskiego 5A, 02-106 Warsaw, Poland

**Keywords:** PSTVd, root, auxin, transcriptome, RNA-seq, microarray

## Abstract

Potato spindle tuber viroid (PSTVd) causes systemic infection in plant hosts. There are many studies on viroid-host plant interactions, but they have predominantly focused on the aboveground part of the plant. Here, we investigated transcriptomic profile changes in tomato roots systemically infected with mild or severe PSTVd variants using a combined microarray/RNA-seq approach. Analysis indicated differential expression of genes related to various Gene Ontology categories depending on the stage of infection and PSTVd variant. A majority of cell-wall-related genes were down-regulated at early infection stages, but at the late stage, the number of up-regulated genes increased significantly. Along with observed alterations of many lignin-related genes, performed lignin quantification indicated their disrupted level in PSTVd-infected roots. Altered expression of genes related to biosynthesis and signaling of auxin and cytokinin, which are crucial for lateral root development, was also identified. Comparison of both PSTVd infections showed that transcriptional changes induced by the severe variant were stronger than those caused by the mild variant, especially at the late infection stage. Taken together, we showed that similarly to aboveground plant parts, PSTVd infection in the underground tissues activates the plant immune response.

## 1. Introduction

Viroids are small plant pathogens that consist of non-capsidated circular, single-stranded, and highly structured RNA molecules without protein coding capability [1,2,3,4]. Members of the family *Avsunviroidae* replicate in the chloroplast through a symmetric rolling-circle mechanism. Potato spindle tuber viroid (PSTVd), which is the type member of the family *Pospiviroidae*, replicates via an asymmetric rolling circle mechanism in the nucleus by utilizing host enzymes such as DNA-dependent RNA polymerase II [5,6], RNase [7], and nuclear DNA ligase 1 [8]. Viroids infect higher plants systemically and can cause various disease symptoms similar to those observed during plant viral infection, including stunting, epinasty, chlorosis, and necrosis or malformation of tubers, flowers, and fruits. In addition to environmental conditions, symptom severity depends on both viroid strain and host plant [9]. 

Considering the non-coding capacity of viroid RNA and the results of extensive research on viroid-host/plant interactions, researchers concluded that viroid pathogenesis can be mediated by interaction of viroid genome itself or by its genome-derived single-stranded (ss)- or double-stranded (ds)-RNA with host proteins and nucleic acids. However, the exact mechanism of viroid pathogenesis is still unclear and many questions remain unanswered.

Implementation of high-throughput methods, including microarray and RNA-seq technology, enable wide transcriptome profiling, which is very helpful in understanding the molecular basis of plant/host-pathogen interactions. Taking advantage of these technologies, there are many studies about leaf transcriptome analysis during viroid infection in various host plants, including PSTVd [10,11,12,13,14], citrus exocortis viroid (CEVd) [15,16,17], citrus viroid III (CVd-III) [18], peach latent mosaic viroid (PLMVd) [19], hop stunt viroid (HSVd) [20,21], chrysanthemum stunt viroid CSVd) [22], hop latent viroid (HLVd) [23,24], and citrus bark cracking viroid (CBCVd) [23,24,25]. Moreover, there is also data about gene expression alterations in PSTVd-infected tubers [26]. In general, these data revealed global transcriptomic changes triggered by viroid infection. Expression of many genes related to photosynthesis, cell wall structure, hormone metabolism and signaling, RNA regulation, protein modification and metabolism, and defense and stress responses, among others, are altered. 

In the natural environment, plants are vulnerable to various pathogens and therefore developed multiple defense mechanisms, including gene silencing, immune receptor signaling, hormone-mediated defense, protein degradation, and regulation of metabolism [27]. Plants may recognize viral effectors with nucleotide-binding leucine-rich repeat (NB-LRR) domain-containing receptors to induce effector-triggered immunity (ETI), and damage/microbe/pathogen-associated molecular patterns (DAMP/MAMPs/PAMPs) via surface-localized pattern recognition receptors (PRRs) to induce PTI (pattern-triggered immunity). PTI is associated with rapid calcium influx, reactive oxygen species (ROS) production, mitogen activated protein kinase (MAPK) phosphorylation cascades, callose deposition, and defense gene expression [28]. Plants also have long-lasting and broad-spectrum induced systemic acquired resistance (SAR) in which endogenous salicylic acid (SA) play a crucial role. Its accumulation results in transcriptional reprogramming of genes that encode pathogenesis-related (PR) proteins [29]. 

To date, wide transcriptomic research on plant-viroid interactions has focused mainly on the aboveground portion of plants (mainly the leaves). Viroids also accumulate in the roots, organs that are crucial for water and nutrient absorption, storage, and anchoring the plant to the ground. Within the rhizosphere, roots are exposed to a vast and diverse microorganism community, some of which are beneficial and some pathogenic. Recently, studies reported that roots have a robust immune system [30,31,32].

Here, we analyzed transcriptional changes in tomato roots (*Solanum lycopersicum* cv. ‘Rutgers’) triggered by mild (M) and severe (S23) PSTVd strains. We examined transcriptomic changes at three infections time points: early (17 days post-inoculation (dpi)), middle (24 dpi), and late (49 dpi). We used two high-throughput methods: (i) microarrays (for changes at 17, 24, and 49 dpi) and (ii) RNA-seq (for changes at 17 dpi). Besides transcriptomic analysis, we evaluated the lignin content in the root cell wall at 24 and 49 dpi. That data indicated that infection with severe PSTVd promoted greater lignification in comparison to mild PSTVd. 

## 2. Materials and Methods 

### 2.1. Plant Growth and PSTVd Inoculation

Tomato seedlings (*Solanum lycopersicum* cv. ‘Rutgers’) with true leaves were mechanically inoculated using carborundum with a 10-µL solution that contained either 2 µg empty pUC9 vector or recombinant pUC9 with cloned infectious cDNA of the severe (S23; GenBank: X76846) or mild (M; GenBank: X76844) PSTVd variant. Plants were grown in individual pots that contained quartz sands (particle size ~1 mm) in a greenhouse with 16 h light at 28–30 °C and 8 h dark at 25 °C. Once or twice per week, the plants were watered with nutrient solution (pH ~6 and electrical conductivity at 2100 µS/cm). The nutrient solution was prepared by adding to 1 L each of the following solutions to 97 L water: (i) macronutrient stock A (g L-1): Ca(NO3)2, 74; KNO3, 19; FeNa-EDTA, 1.2; (ii) macronutrient stock B (g L-1): K2SO4, 72; MgSO4, 60; H3PO4, 15.3 mL L-1; and (iii) micronutrient supplement (mg L-1): FeSO4, 900; MnSO4, 307; H3BO3, 200; CuSO4∙7H2O, 78; ZnSO4, 120; (NH4)6Mo7O24∙4H2O, 50. 

### 2.2. Lignin Quantification

Protein-free cell wall was prepared according to the method described by [33,34]. Briefly, dry roots were homogenized in a mortar with 50 mM potassium phosphate buffer (pH 7.0). After centrifugation (2000× *g*, 5 min), the pellet was washed several times with: (i) phosphate buffer (pH 7.0), (ii) 1% (*v*/*v*) Triton X-100 in phosphate buffer (pH 7.0), (iii) 1 M NaCl in phosphate buffer (pH 7.0), (iv) distilled water, and (v) acetone. The obtained dry matter (after overnight incubation at 60 °C) was defined as the protein-free cell wall fraction. The lignin content was determined using the acetyl bromide method [34,35]. Five mg protein-free cell wall fraction were incubated with 0.5 mL 25% acetyl bromide (*v*/*v* in glacial acetic acid) at 70 °C for 30 min. Next, the samples were cooled on ice and mixed with 0.9 mL 2 M NaOH and 0.1 mL 7.5 M hydroxylamine-HCl. The volume was adjusted to 10 mL with glacial acetic acid, centrifuged (2000× *g*, 5 min), and the absorbance was measured at 280 nm. A standard curve with lignin (alkali; Sigma-Aldrich 370959) was generated, and the absorptivity (ε) value obtained was 21.4 g-1 L cm-1. The results are expressed as mg lignin g-1 cell wall. The data are expressed as the mean of three independent experiments ± standard deviation. 

### 2.3. RNA Extraction

Whole roots were collected at 17, 24 and 49 dpi and homogenized in a mortar with a pestle and liquid nitrogen. Total RNA was isolated using the RNeasy Plant Mini Kit with on-column DNA digestion (Qiagen, Hilden, Germany), following the manufacturer’s protocol. Additionally, digestion with TURBO™ DNase (Ambion, Austin, TX, USA) was applied to remove DNA traces. RNA quality and integrity were evaluated using a Bioanalyzer 2100 (Agilent Technologies), and the concentration was estimated using a Nano Drop ND-1000 spectrophotometer.

### 2.4. Northern Blot Hybridization

Equal amounts of total RNA (2 µg) isolated from individual roots were separated on a 5% polyacrylamide gel (19:1 acrylamide:N,N’-methylenebisacrylamide) with 8 M urea in Tris-borate-EDTA (TBE) buffer. The separated RNA was transferred to Hybond N+ nylon membranes (Roche, Mannheim, Germany) using a Yrdimes semidry transfer system (Wealtec Corp., Meadowvale Way Sparks, NV, USA) and crossed-linked by irradiation with 0.14 J/cm2 UV light. Hybridization and detection was performed using the DIG Northern Starter Kit (Roche), according to manufacturer’s instructions, with a PSTVd-specific digoxigenin-labeled RNA probe (DIG RNA labeling mix; Roche, Mannheim, Germany).

### 2.5. PSTVd Sequencing

Reverse transcription polymerase chain reaction (RT-PCR), direct sequencing of PCR products, and data analysis was performed as described previously [14]. 

### 2.6. Transcriptome Profiling Using Microarrays

Microarray analysis was performed in Corelab (Available online: www.corelab.pl), where Affymetrix technology (https://www.thermofisher.com) and Partek Genomics Suite Software are routinely used. Three biological replicates (three roots) for each treatment (S23, M, or empty pUC9 vector) from each time point (17, 24, or 49 dpi) were processed independently, except that only two roots from S23-infected plants at 17 dpi were analyzed. Analysis was performed using the GeneChip^®^ Tomato Genome 1.0 ST Array (Affymetrix, Santa Clara, CA, USA). All steps were performed as described previously [14]. Affymetrix probes were aligned to the released version of the tomato genome cDNA sequence ITAG 3.2 (Available online: http://solgenomics.net). Statistical analysis of microarray data was performed as described previously [14] using Partek Genomic Suite v 7 sofware with the use of RMA (robust multiarray averaging). 

The complete datasets of the microarray experiment are available in the NCBI Gene Expression Omnibus (GEO) database repository with accession number GSE111736.

### 2.7. Transcriptome Profiling by RNA-seq

RNA-seq analysis was performed in Corelab (Available online: www.corelab.pl). ERCC RNA Spike-In Mix 1 (Thermo Fisher Scientific) was added as an internal control to each sample. Ribosomal RNA (rRNA) was removed using Ribo-Zero Plant rRNA Removal Kit (Illumina). Libraries were prepared with Ion Total RNA-seq Kit v2 and Ion Xpress RNA-seq Barcode 1-16 Kit, according to user guide. Sequencing template was generated with Ion PI™ Template OT2 200 Kit v3 using an Ion OneTouch™ 2 System. Sequencing was performed on an Ion PI™ chip v2 and Ion Proton™ sequencer using Ion PI™ Sequencing 200 Kit v2 (all Ion Torrent kits and software are trademarks of Thermo Fisher Scientific).

Base calling and adapter trimming was performed automatically by Torrent Suite software. Residual rRNA and ERCC reads were identified and removed using bbsplit and filterbyname scripts from BBTools suite (Brian Bushnell). Reads were aligned to the ITAG3.2 genome using TMAP 5.0.13, with soft clipping from both ends and set to return all the mappings with the best score. Other settings followed Torrent Suite defaults. Unaligned reads were aligned with BBMap (Brian Bushnell). Quantitation to ITAG3.2 transcripts and differential expression analysis was performed in Partek Flow (Partek Inc.) using the Partek GSA algorithm. 

The complete datasets of the microarray experiment are available in the NCBI Gene Expression Omnibus (GEO) database repository with accession number GSE125228.

### 2.8. Functional Annotation of Genes and Pathway Analysis

Gene Ontology (GO) enrichment analysis of differentially expressed genes (DEGs) that exhibited at least two-fold change (FC) and a *p*-value < 0.05, was performed using Blast2GO software [36] with Fischer’s exact test (corrected for multiple testing). GO terms with a false discovery rate (FDR) < 0.05 were considered to be statistically significant.

Pathway annotation was performed using the Kyoto Encyclopedia of Genes and Genomes (KEGG) via the KEGG Automatic Annotation Server (KAAS, Available online: http://www.genome.jp/kegg/kaas/; [37]. The KOBAS software [38] was used to determine the statistical enrichment of DEGs in KEGG pathways. KEGG terms with a corrected *p*-value < 0.05 were considered to be statistically significant. MapMan software [39]; https://mapman.gabipd.org/) was used for pathway visualization of DEGs involved in the PSTVd-plant interaction.

Transcription factors (TFs) and protein kinases (PKs) were identified based on the data deposited at http://bioinfo.bti.cornell.edu/cgi-bin/itak/index.cgi [40]; PK classification was according to [41].

The Venny 2.1.0 online tool (Available online: http://bioinfogp.cnb.csic.es/tools/venny/index.html) was used to create Venn diagrams [42].

### 2.9. NanoString nCounter Analysis

NanoString nCounter multiplex gene expression analysis (NanoString Technologies Inc., Seattle, USA; www.nanostring.com) at the Core Facility Molecular Biology, Medizinische Universität Graz [43,44] was performed to evaluate the level of the selected transcripts and to verify the PSTVd (+) RNA level. An nCounter CodeSet designed for viroid RNA and 3 genes are shown in Supplementary Table S1. For gene expression normalization of the selected DEGs and PSTVd titer, the geometric mean of the expression of three housekeeping genes, namely *alpha glucosidase II* (*gluII*, NM_001247101.2), *glyceraldehyde 3-phosphate dehydrogenase* (*GAPDH*, NM_001247874.2), and *actin* (NM_001321306.1), were used. 

### 2.10. Statistical Analysis

Statistical analysis was performed with Statistica v.12 Software (StatSoft Inc., USA). The results are presented as the mean of three replicates ± standard deviation (SD). Differences were regarded as statistically significant if *p* < 0.05.

## 3. Results

### 3.1. Mild and Severe PSTVd Infection in Tomato Roots

Inoculation of tomato plants with PSTVd-S23 and PSTVd-M leads to development of systemic infection with typical severe and mild symptoms, respectively.

**Figure 1:**
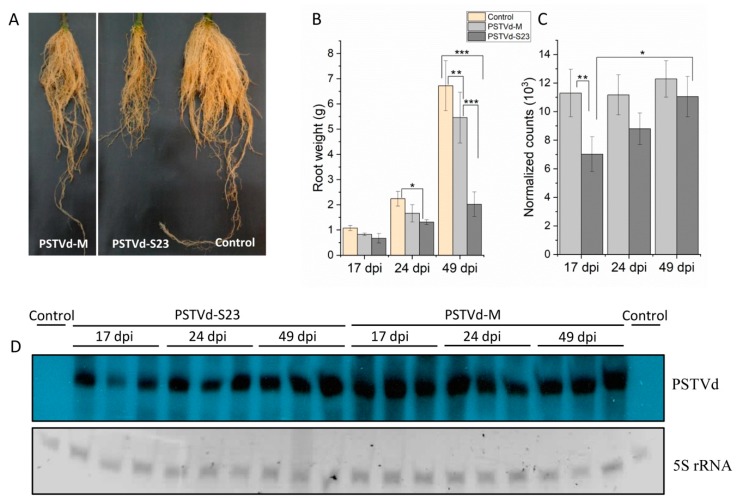
Potato spindle tuber viroid (PSTVd) infection in tomato roots. (**A**) Root comparison at 49 dpi. (**B**) Root weight at the indicated time points. Each bar represents the arithmetic mean of the weight of three roots with the standard deviation indicated. (**C**) Relative levels of viroid RNA as estimated using the Nanostring nCounter method and nSolver analysis software. The data is presented in arbitrary units compared to control plants. (**D**) Northern blots that show the presence of PSTVd in infected roots at the indicated time points. Two µg total RNA were separated in 5% polyacrylamide gel with 8 M urea. One-way analysis of variance followed by Tukey’s honest significant difference test was used to determine statistically significant differences. * *p* < 0.05; ** *p* < 0.01; *** *p* < 0.001.

Whole roots for RNA isolation were harvested at 17, 24, and 49 dpi. At the late stage, there were large differences in root growth and development between plants infected with the mild and severe variants (Figure 1A,B). The levels of PSTVd RNA in the infected roots were verified by two methods: Northern blots and Nanostring nCounter analysis. Northern blots indicated a higher level of PSTVd-M than PSTVd-S23 at earlier stages of infection (17 and 24 dpi) (Figure 1D). Nanostring nCounter analysis confirmed these results; however, the observed difference was only statistically significant at 17 dpi (Figure 1C). The presence of the original M and S23 variant sequences was confirmed in root samples (from 24 and 49 dpi) by sequencing the synthesized RT-PCR products. 

### 3.2. Overview of Gene Expression in Roots during PSTVd Infection

Using microarray technology, we performed root transcriptome profiling at 17, 24 and 49 dpi using mock-inoculated, PSTVd-S23- and PSTVd-M-infected plants. Root samples collected at 17 dpi were also subjected to RNA-seq analysis.

#### 3.2.1. Microarray

**Figure 2:**
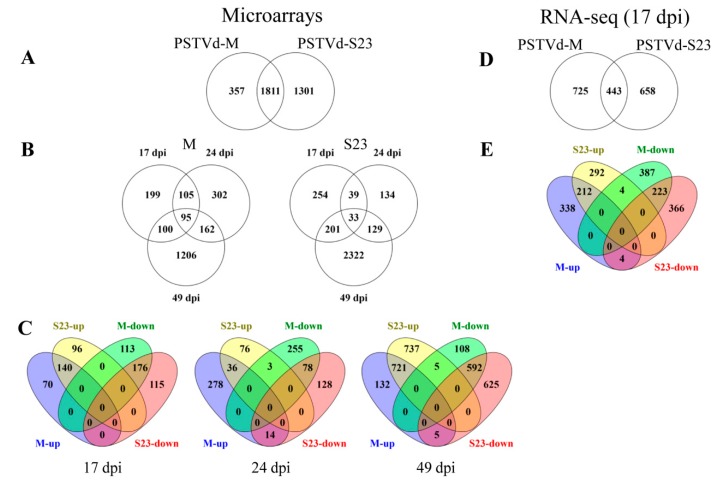
The number of differentially expressed genes (DEGs) identified in microarray (**A**–**C**) and RNA-seq analysis (**D**,**E**). Venn diagrams display the distribution of: (**A**) Total DEGs in M- and S23-infected roots, (**B**) and at 17, 24 and 49 dpi. (**C**) Up- and down-regulated genes observed at the indicated time points. (**D**) Total DEGs at 17 dpi. (**E**) Up- and down-regulated genes at 17 dpi.

In total, 3469 genes were significantly altered (*p* < 0.05 and FC ≤ −2 or ≥ 2) in viroid-infected plants for at least one of the three time points (Table S2; Figure 2 A–C). In both infections, the highest number of DEGs occurred at 49 dpi. Only 95 and 33 genes were consistently altered at all three time points during mild and severe infection, respectively. Based on functional analysis performed in MapMan, for both infections, most of these genes are related to RNA regulation, processing and binding, protein metabolism and modification, and miscellaneous enzymes. 

#### 3.2.2. RNA-seq

High-throughput RNA-seq generated 16.26–25.70 million raw reads per sample; three biological replicates were performed for each treatment (PSTV-M, PSTVd-S23, and mock-inoculated plants). After cleaning, 15–23.74 million cleaned reads for each sample were obtained and mapped to the ITAG3.2 genome (Table S3). 

Table S4 contains the complete list of transcripts (5437) with the cutoff *p* < 0.05. Using the same criteria for *p*- and FC values as for microarray analysis, 1168 and 1101 genes were differentially regulated during mild and severe infection, respectively (Figure 2D,E; Table S4). Only eight DEGs were regulated in the opposite direction; intriguingly, most of them were related to the stress response. Four genes (Solyc06g008265, Solyc04g014400, Solyc06g060710, and Solyc01g066457) that were down-regulated by M but up by S23 encoded two leucine-rich repeat (LRR) receptor kinases, nucleotide-diphospho-sugar transferase, and epoxide hydrolase-like protein, respectively. Another four genes (Solyc03g097420, Solyc08g007070, Solyc09g005420, and Solyc11g011600), which encode HVA22-like protein, disease resistance protein RPM1, major latex protein (MLP), and GRAM domain protein/ABA-responsive-like protein, respectively, were induced in M infection but repressed in S23. The last of these genes was very strongly down-regulated (FC ~ -609). 

We also selected the top 30 genes that were most up- or down-regulated by each PSTVd variant. Some of the down-regulated DEGs in M-infection encode pentatricopeptide repeat (PPR)-like superfamily proteins that are involved in RNA editing, BED zinc finger, lipoxygenase associated with jasmonic acid (JA) metabolism, RING/U-box and F-box family proteins related to protein degradation, AT-rich interactive domain protein involved in RNA regulation of transcription, disease resistance protein, and others. Interestingly, this group contained mainly DEGs unique for M infection. The most strongly repressed genes in S23 infection encoded TFs from MYB and basic helix-loop-helix (bHLH) families, carotenoid isomerase, flagellar biosynthesis protein, and DNA-directed RNA polymerase subunit beta, among others. One third of this group of genes was also regulated by the M variant. With regards to the highly up-regulated genes, half of them overlapped in both infections. Among them were genes that code for a bHLH-family TF, transcriptional co-repressor, glyoxylate reductase, NAD kinase, calcium-transporting ATPase, and chlorophyll a-b binding proteins, among others.

### 3.3. KEGG Pathways Influenced by the Infection Time Course

Genes that were up- or down-regulated during mild or severe infection at particular time points (based on microarray analysis) were subjected to KEGG enrichment analysis; a short list of the enriched pathways is reported in Table 1, while all data appears in Supplementary Table S5. Over twice as many pathways were enriched in the up-regulated compared to the down-regulated gene set. Some pathways were common for M and S23 variants at the same time point, for example, alpha-linolenic acid metabolism and steroid biosynthesis at 17 and 49 dpi, respectively. However, several pathways were only affected in S23 at 49 dpi. Over 70% of the enriched pathways were related to metabolism, for example, ‘Biosynthesis of secondary metabolites’ or ‘Cysteine and methionine metabolism’. Of the 10 pathways that were not correlated to metabolism, four (‘Ribosome’, Spliceosome’, ‘Protein processing in endoplasmic reticulum’, and ‘Proteasome’) relate to genetic information processing, another four (‘Phagosome’, ‘Regulation of autophagy’, ‘Peroxisome’, and ‘Endocytosis’) to cellular processes, one (‘Plant-pathogen interaction’) to organism systems, and one (‘Plant hormone signal transduction’) to environmental information processing. With the exception of two pathways ‘Regulation of autophagy’(down-regulated) and ‘Plant hormone signal transduction’ (variously regulated) ), the 8 other were up-regulated.

### 3.4. Enriched GO Terms Related to Biological Process over the Infection Time Course

**Figure 3:**
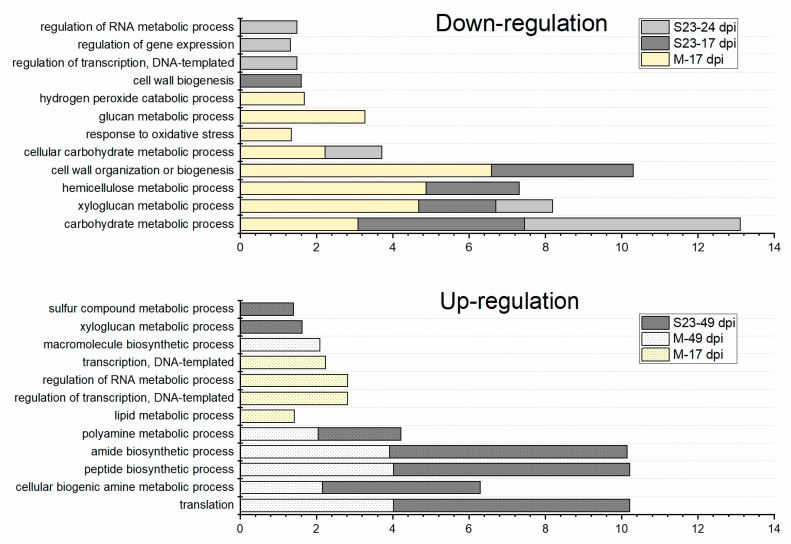
Enriched Gene Ontology (GO) terms in the biological processes (BP) category for the down- and up-regulated DEGs at the indicated infection time point. Only selected categories are presented. No GO term was enriched at 24 and 49 dpi for up- and down-regulated DEGs, respectively. X-axis represents the value of log_10_(1/FDR); enrichment false discovery rate (FDR) value < 0.05 was used as a cutoff.

To better understand which biological processes (BP category) were affected in roots infected with mild or severe PSTVd at the successive time points, we performed GO enrichment analysis (Figure 3 and Table S6). At 17 dpi, most of overrepresented GO terms in down-regulated genes were common to both infections, for example, cell wall organization or biogenesis. The category ‘response to oxidative stress’ was one of several categories distinguished only in M infection. At 24 dpi, enriched GO terms in S23 infection included those related to regulation of biological processes (including RNA biosynthesis) and those associated with metabolic processes like cellular carbohydrate metabolic process. 

GO terms mainly associated with transcription regulation were enriched in a set of genes up-regulated by M infection at 17 dpi. Intriguingly, some of these categories were already observed in a set of genes down-regulated by S23 at 24 dpi. No enriched GO terms were identified in the group of genes upregulated by S23 at 17 or 24 dpi by either variants. The ‘xyloglucan metabolic process’ that was overrepresented in M- and S23-down-regulated genes was identified in genes up-regulated after S23 infection at 49 dpi. Most of enriched categories at 49 dpi were common for both infections (Figure 3). 

Overall, these data demonstrate overrepresented GO terms related to cell wall biogenesis, organization, and modification were repressed by both PSTVd variants during early infection stages. Comparatively, those related to translation, including such categories as amide biosynthetic process and peptide metabolic process, were activated by these variants during late infection. 

### 3.5. Analysis of DEGs Identified by RNA-seq Analysis

DEGs identified from RNA-seq at 17 dpi were also subjected to gene enrichment analysis (Table S7). Many of the same GO terms, namely those related to cell wall organization, modification, and degradation, were repressed in both infections. Another common enriched category was ‘response to stress’, which includes genes that encode peroxidases, defensins, and MLP-like proteins. The GO term ‘oxidation-reduction process’, which contains genes encoding cytochrome P450s, alcohol or aldehyde dehydrogenases, defense-peroxidase CEVI1, and lipid desaturase-like protein (CEVI19), was one of three GO terms exclusively repressed by the M-variant infection. Cell-wall-related categories, including ‘glucan metabolic process’ and ‘external encapsulating structure organization’, were observed only in S23 infection.

With regards to up-regulated DEGs, GO enriched terms were indicated only in S23 infection. The observed categories included ‘protein modification process’, ‘phosphorylation’, ‘multi-multicellular organism process’, or ‘reproduction’, which are predominantly related to genes that encode serine/threonine-protein kinases, receptor-like protein kinases or MAP kinase kinase kinase (MAPKKK), RING/U-box superfamily proteins, and U-box domain-containing proteins.

We also examined DEGs that were exclusively up- or down-regulated by only one of the PSTVd variants (Tables S4 and S7). The set of S23-specific down-regulated genes, included one category, ‘carbohydrate metabolic process’, which comprises genes related to cell wall degradation and biosynthesis such as *Cel2*, *glycosyl hydrolase*, pectin lyase, and *cellulose synthase*. Different categories were enriched by up-regulated genes from M and S23 infection. The M variant featured enriched categories related to gene expression, namely TFs from the AP2/ER, basic leucine zipper (bZIP) domain, C2C2, HB, and WRKY families, while the S23 variant enriched genes related to protein modification. 

### 3.6. Expression of Hormone-Related DEGs

**Figure 4:**
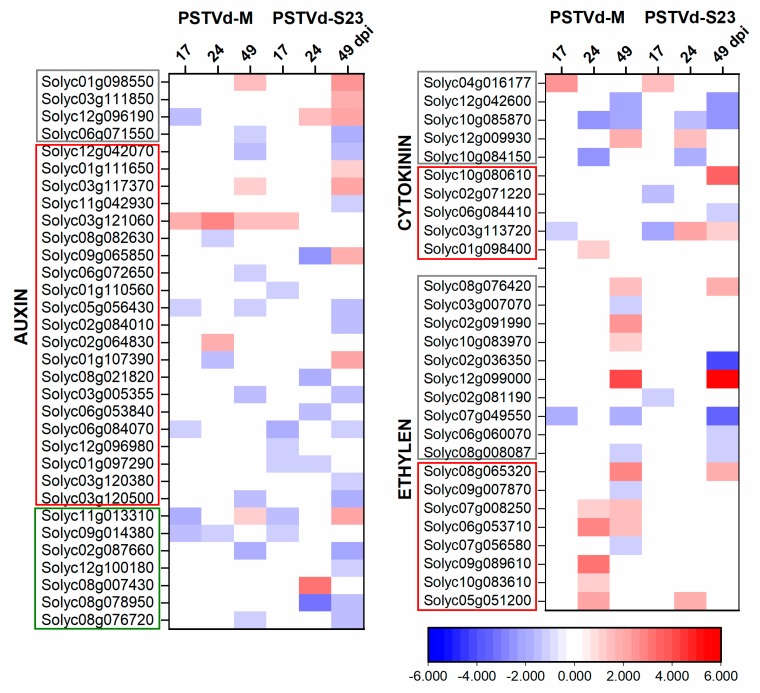
Heat map of log_2_ FC of DEGs related to plant hormones. DEGs involved in biosynthesis, signaling and transport are in grey, red, and green frames, respectively.

We observed changes in many transcripts related to hormone biosynthesis, transport, and signaling (Figure 4, Table S8). Auxin is the dominant regulator of lateral root development. Root-generated auxin helps maintain the gradients and maxima required for normal root development [45]. Both microarray and RNA-seq indicated on down-regulation of many genes related to auxin biosynthesis, signaling, and transport. More DEGs were observed during severe infection, especially at 49 dpi (Figure 4). 

Contrary to auxin, cytokinins (CKs) negatively regulate lateral root formation. Genes related to CK biosynthesis were similarly regulated in both infections (Figure 4). In CK signal transduction, one of the two *histidine-containing phosphotransfer protein (AHP)* genes was up-regulated in M and another was down-regulated in S23 infection. AHPs transmit phosphorylation signals to response regulators (ARRs), which act as transcription factors [46]. Type-A ARRs (Solyc10g079595 and Solyc02g071220) act as negative regulators for the signal transduction pathway; they were down-regulated at 17 dpi. Moreover, the gene *KMD3* (*KISS ME DEADLY*), which encodes another negative regulator of the CK signaling pathway [47], was highly up-regulated at 49 dpi in S23 infection.

RNA-seq analysis confirmed down-regulation of CK signaling pathway genes in both infections and differential regulation of genes related to CK biosynthesis. One of these genes (Solyc06g075090) encodes cytokinin riboside 5’-monophosphate phosphoribohydrolase and was highly up regulated in M infection (FC = 523), while another one (Solyc04g016220) that encodes cis-zeatin O-glucosyltransferase was highly down-regulated (FC = −244) in S23 infection.

Genes encode three enzymes, namely S-adenosyl-L-methionine synthetase (SAM1), 1-aminocyclopropane-1-carboxylate (ACC) synthase (ACS), and ACC oxidase (ACO), from the ethylene (ET) biosynthesis pathway were altered prominently by both infections at 49 dpi (Figure 4, Table S8). In the signal transduction pathway, two-times more genes were altered in M compared to S23 infection (all but two were up-regulated). RNA-seq indicated ET biosynthesis repression in both infections and activation of signal transduction pathway.

The expression of most genes that encode enzymes from the gibberellin (GA) biosynthesis pathway, such as ent-kaurenoic acid oxidase (KAO), GA20-ox, and GA3-ox, as well as those from the signaling pathway, including DELLA and GID1 proteins, were decreased at 49 dpi in both infections. RNA-seq indicated down-regulation of biosynthesis and signaling at 17 dpi in M-infection (Table S8).

During both infections, expression of many ABA biosynthesis and signaling genes were differentially regulated depending on the stage of infection. RNA-seq recognized many variably regulated genes involved in ABA transport upon viroid infection (Table S8).

Independent of the PSTVd strain, only a few brassinosteroid (BR)-related genes were altered at 17 and 24 dpi. Contrarily, at 49 dpi, there were significantly more DEGs related to the BR biosynthesis and signaling pathways (especially in S23 infection) and their expression was elevated During both infections, several genes involved in JA biosynthesis and signaling were induced; only a few genes were down-regulated. RNA-seq indicated that at 17 dpi more genes associated with both biosynthesis and signaling were regulated in mild than in severe infection, *LOX3*, which encodes lipoxygenase, was strongly down-regulated (FC = −116), but only by the M variant. 

We also observed altered expression of genes encoding proteins belonging to salicylic acid (SA) pathway. Both, microarray and RNA-seq indicated on up-regulation of genes that encode regulatory NPR1/NIM1 and PR1 proteins at 17 dpi. Contrarily, at 49 dpi genes that encode TGAs TF from bZIP-family, were down-regulated (Table S8).

### 3.7. DEGs That Encode TFs and PKs

TFs and PKs play significant roles in plant growth and development regulation and response to biotic and abiotic stimuli [40,48,49,50,51].

Overall, in microarray analysis, more than 200 DEGs that encode TFs and 100 DEGs that encode PKs were recognized in the infected roots during PSTVd infection. These TFs belong to many families, including AP2/ERF, bHLH, NAC, bZIP, and WRKY, all of which play important roles in plant defense. Microarray and RNA-seq indicated that approximately 15% of all known tomato WRKY genes were elevated in our study (Table S9). RNA-seq allowed us to recognize more TFs, mostly from the bHLH, MYB, MYB-related, and GRAS families. Similarly, more PK-encoding genes were identified by RNA-seq at 17 dpi than by microarrays, for example, three genes that encode calcium-dependent protein kinases (CDPKs; Table S9).

### 3.8. PSTVd Infection Repressed the Expression of Cell-Wall-Related Genes during Early Infection

We observed altered expression of many genes related to cell wall synthesis and remodeling, including genes that encode cellulose synthases, endo-1,4-β-glucanases, COBRA-like proteins, pectin esterases, expansions, arabinogalactan-proteins, and proline-rich proteins (Table S10). Genes associated with degradation (e.g., *pectate lyases* and *polygalacturonases*) were also regulated. In both infections at 17 and 24 dpi, there were many more down-regulated genes, but at 49 dpi, the percentage of up-regulated genes grew significantly. *AGP1* (Solyc02g092790), which encodes arabinogalactan, and *BR1* (Solyc09g092520), which encodes BR-regulated tomato xyloglucan endotransglucosylase/hydrolase (XET), shifted from down- to up-regulated. Down-regulation of cell-wall related genes by both PSTVd variants was confirmed by RNA–seq.

### 3.9. Expression of Lignin-Biosynthesis-Specific Genes and Lignin Quantification

The phenylpropanoid pathway is involved in the synthesis of numerous secondary metabolites, including lignin, iso-flavonoid-phytoalexins, and other phenolic compounds [52]. 

**Figure 5:**
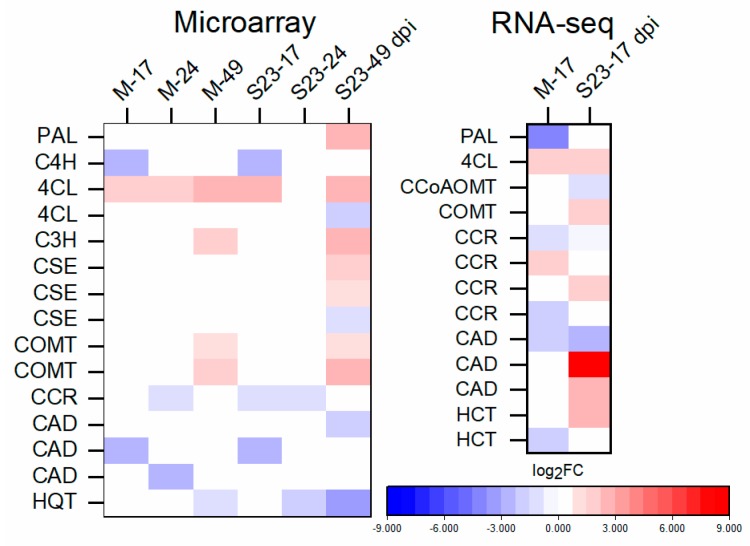
Heat map of log_2_ FC of main genes related to lignin biosynthesis identified by microarray and RNA-seq.

Microarray results demonstrated genes that encode enzymes specific for lignin biosynthesis, and those involved in the general phenylpropanoid pathway, were mostly down-regulated at 17dpi in both M- and S23-infected plants (Figure 5, Table S11). At later infection stages, the changes were variable. The *4-coumarate:CoA ligase* (*4CL*) transcript level (encoded by Solyc12g094520), which catalyzes the last step in the general phenylpropanoid pathway, was higher in infected compared to control plants starting from 17 dpi. Additionally, RNA-seq analysis showed that three other genes from the lignin specific pathway, namely *cinnamoyl-CoA reductase (CCR)* and two *cinnamyl alcohol dehydrogenase (CADs)*, were up-regulated in S23 infection. In *Arabidopsis*, *CAD-C* and *CAD-D* are the primary genes involved in lignin biosynthesis [53]. At 49 dpi, phenylalanine ammonia-lyase (PAL)- and caffeoyl shikimate esterase (CSE)-encoding genes were up-regulated in S23-infected roots. *p-coumarate 3-hydroxylase (C3H)* and *caffeic acid O-methyltransferase (COMT)* were up-regulated in both S23- and M-infected roots. Moreover at 49 dpi, some of the genes that encode peroxidases, which catalyze the last step of lignin biosynthesis, were up-regulated. 

**Figure 6:**
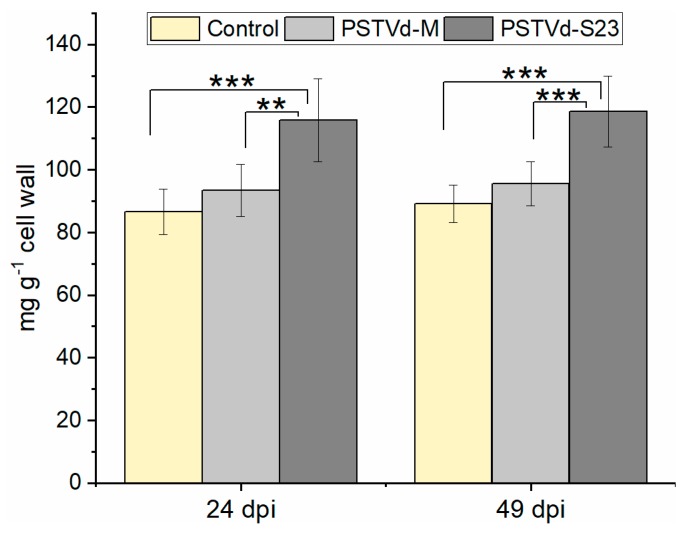
Lignin content in roots at 24 and 49 dpi. One-way analysis of variance, followed by Tukey’s honest significant difference test, was performed to determine the significance of the differences; ** *p* < 0.01, *** *p* < 0.001. Each bar represents the arithmetic mean of the lignin amount from three roots with the standard deviation indicated.

In addition to transcript analysis, we measured the lignin content in the root cell wall at 24 and 49 dpi. In S23-infected roots, there was a moderate but statistically significant (*p* < 0.001) higher lignin content compared to both M-infected and mock-inoculated plants (Figure 6).

### 3.10. Up-Regulation of Proteasome-Related Genes

Protein degradation by the ubiquitin-proteasome system (UPS) participates in many cellular processes, where it regulates plant growth and development, senescence, embryogenesis, hormonal signaling, plant response, and immunity [54,55]. The highest number of DEGs related to ubiquitin-dependent degradation was observed at 49 dpi. RNA-seq analysis revealed that at 17 dpi most ubiquitin-related genes were up-regulated (mostly by the M variant), data that are consistent with the microarray results (Table S12). 

### 3.11. Up-Regulation of DEGs Related to Plant-Pathogen Interaction

**Figure 7:**
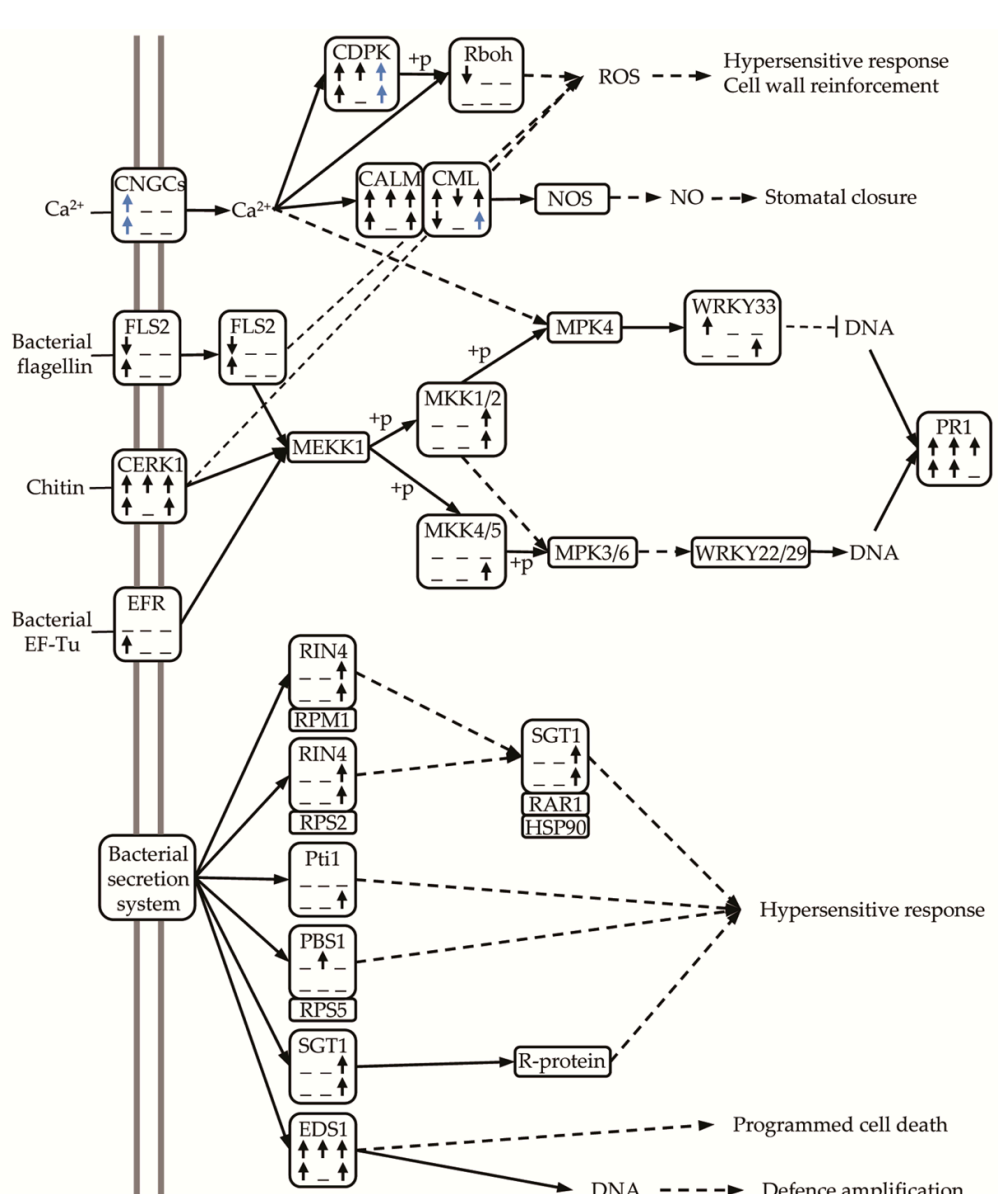
Plant-pathogen interaction pathway. The results are based on KEGG KASS analysis [37] of all DEGs identified in microarray and RNA-seq. Arrows indicate that all or most (blue arrow) DEGs encoding the particular pathway component are up- or down-regulated. DEGs from PSTVd-M and PSTVd-S23 infections are presented in the first and the second row, respectively.

In the plant-pathogen interaction pathway (ko04626) altogether (from microarray and RNA-seq) 34 and 38 DEGs were identified in M, and S23 infection, respectively. Except genes encoding flagellin-sensing 2 (FLS2) and respiratory burst oxidase (Rboh) repressed at 17 dpi in mild infection, and calcium binding protein (CML) at 17 dpi (S23 infection), and 24 dpi (M infection), the most of other DEGs were up-regulated (Figure 7). In PTI pathway, genes encoding membrane receptors such as: chitin elicitor receptor kinase 1 (CERK1) and EF-Tu Receptor (EFR), and those encoding components of mitogen-activated protein kinase cascade (MKK1/2, MKK4/5), WRKY33, as well as those link to cytosolic Ca^2+^ (the cyclic nucleotide gated channel (CNGC)) were up regulated. Similarly, many genes from ETI pathway were up-regulated at different stages of mild and severe infection, for example those encoding suppressor of G2 allele of SKP1 (SGT1), enhanced disease susceptibility 1 protein (EDS1), RPM1-interacting protein 4 (RIN4), or serine/threonine-protein kinase PBS1 (Figure 7).

### 3.12. Validation of Microarray and RNA-seq

**Figure 8:**
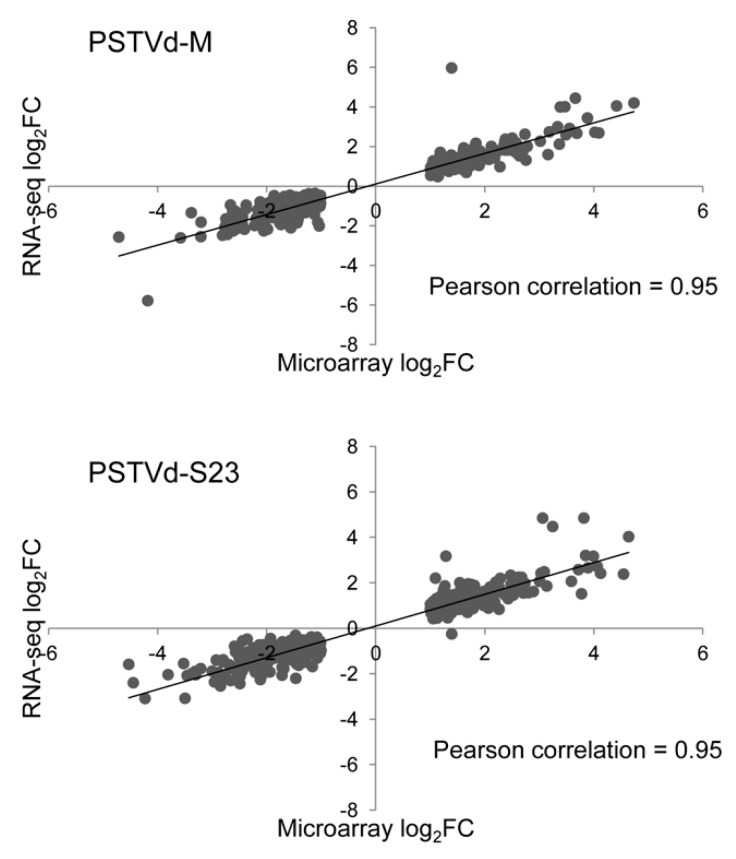
Correlation of microarray and RNA-seq data for DEGs identified by both analysis.

**Figure 9:**
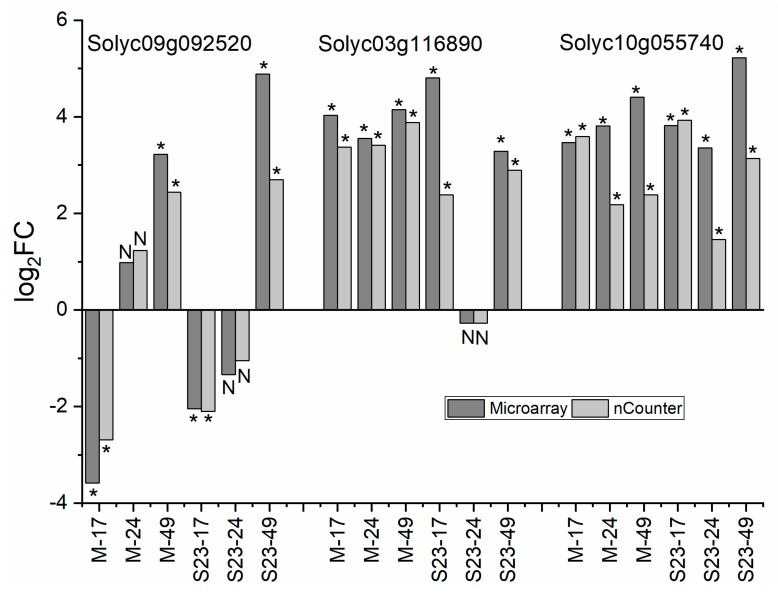
Validation of microarray results by Nanostring nCounter analysis. * *p* < 0.05; N, not statistically significant.

Correlation between microarrays and RNA-seq for DEGs that were common for both methods was high; the Pearson correlation coefficient was approximately 0.95 for each PSTVd variant (Figure 8). Additionally, 3 genes related to cell wall, amino acid transport, and TF that were classified as DEGs for at least at one time point in both infections were selected for nCounter analysis. The mRNA level of each of these selected genes was assayed in samples from both variants collected at each time point regardless of whether the gene was classified as differentially expressed at this time point. The results demonstrated that expression profiles of these selected genes were consistent with those determined by microarray (Figure 9).

## 4. Discussion

In this study, we employed two high-throughput transcriptomic approaches. The first, microarray, is a well-established, reliable system with the potential to provide a rapid overview of transcript level variations. Microarrays are relatively inexpensive compared to RNA-seq. Nevertheless, RNA-seq has many advantages, namely higher gene coverage and increased sensitivity in gene expression monitoring [56]. As expected, RNA-seq identified more transcripts than microarrays (Figure 2). The Pearson correlation coefficient between microarray data (genes classified as DEGs) and corresponding RNA-seq data was estimated at 0.81 and 0.79 for M and S23, respectively (Figure S1); these values are considered to represent a high level of correlation [57]. Our goal was not to compare the two methods, but rather to determine whether some DEGs recognized by microarray are not indicated by RNA-seq due to lack of statistical significance or low expression changes. Other studies concluded that these two methods complement each other, for example, in a study of the *Chlamydomonas reinhardtii* hydrogen production process [56], transcriptome profiling of wild-type and *hrpX* mutant strains of the *ƴ-Proteobacterium Xanthomonas citri* [58], and *Mesorhizobium huakuii* 7653R that occurs symbiotically with a host plant (*Astragalus sinicus*) or as free-living cell in the soil [59]. Moreover, comparative analysis of gene expression in two zones of the *Arabidopsis* root apex relevant to spaceflight showed how genes with low expression levels can be missed by RNA-seq analysis performed at a generally accepted read depth [60].

We analyzed transcriptomic changes in tomato roots during infection with two PSTVd variants with significantly different virulence. The extent of transcriptomic changes depended on the stage of infection as well as on the viroid strain (Figure 2, Tables S2 and S4). In parallel we compared PSTVd-M and PSTVd-S23 RNA levels by Northern blots and Nanostring nCounter (Figure 1C,D). Contrary to PSTVd-M, level of PSTVd S23 RNA was not stable. Wang and coauthors [61] reported that *TFIIIA* suppression and overexpression in *Nicotiana benthamiana* is correlated with decreased and increased PSTVd replication, respectively. Both microarray and RNA-seq indicated no altered *TFIIIA* at 17 dpi; however, at 49 dpi it was up-regulated by S23 infection. This finding corresponds with the observed increase of viroid RNA (Figure 1C, D).

A study of tomato spotted wilt virus (TSWV) systemic infection on tomato shoots and roots revealed organ-specific transcriptional responses [62]. Organ specific differences in phytohormone and antioxidative responses upon PSTVd infection in potato leaves and tubers were reported recently [63]. Comparison of the present study with our previous microarray analysis concerning the leaf transcriptome also revealed some differences. First of all, the observed differences between the mild and severe strains in infected roots were lower than those observed in leaves. This finding applies to the total number of commonly regulated genes as well as to those regulated at particular stage of infection or unique for one variant. In both plant organs, we observed changes in the expression of genes that encode MAPKs, WRKY TFs, CDPKs, NBS-LRRs, PR proteins, receptor-like kinases (RLKs), and others from plant-pathogen pathway. This data indicate the activation of the plant immune response (Figure 7), which was reported earlier in many viroid studies focused on leaf transcriptome, for example, PSTVd [13], CEVd [16,17], or CBCVd and HLVd [24]. In our studies, for example, there are subtle differences in expression of *CERK1*, *FLS2*, *EDS1*, or *CDPK*. For example, in roots, *CDPK*s expression was altered mostly at 49 dpi. Two *CDPK*s recognized by microarray and RNA-seq were down-regulated, at 49 and 17 dpi, respectively while others (7 from both methods) were up-regulated. The similar number of *CDPK*s were changed in PSTVd-infected leaves [14], and four of them are common for both organs but regulated at other stages of infection development.

We observed some differences in DEGs related to hormone metabolism and signaling. SA is involved in the basal resistance of tomato plants to CEVd and TSWV. Infection of SA-deficient *NahG* plants with CEVd and TSWV dramatically increase early diseases symptoms. Moreover, earlier and more intense accumulation of hydroxycinnamic amides, ethylene and defense–related proteins such as PR1 and P23 occurs in *NahG* plants compared to plants without the *NahG* transgene. Application of benzothiadiazole (BTH), which activates the SAR pathway downstream of SA signaling, improves the resistance of *NahG* plants to these pathogens [64]. In both roots and leaves, only one SA-biosynthesis-related gene (*PAL*) was regulated (only by S23). However, in leaves, it was down-regulated at 17 dpi but up-regulated at 49 dpi in roots. The signal transduction pathway was regulated in both S23- and M-infected roots; however, in leaves this pathway was mostly modulated upon S23 infection, and only up-regulation was observed. In roots, regulation of the ET biosynthesis and signaling pathways mostly occurred upon mild infection while in leaves upon severe infection.

In general, JA was up-regulated, but in leaves this change was stronger after S23 infection at all stages. Comparatively, the change in roots was stronger after M infection. GA was in general up-regulated at 24 dpi in S23-infected leaves, but in roots infections with both strains down-regulated these genes (mostly at 49 dpi). BRs play an important role in modulating the trade-off between plant growth and defense. During MTI, genes related to BR biosynthesis are down-regulated because BR signals can prioritize growth over immunity [65]. Regulation of BR-related genes in leaves occurs almost exclusively in severe infection, and at 17 and 49 dpi these genes were down-regulated. Comparatively, in the roots, the DEGs were down-regulated at 17 dpi but up-regulated at 49 dpi for both infections. 

*CEVI-1* was overrepresented in the GO category of DEGs down-regulated by M variant. As reported by Vera and colleagues [66], four weeks after CEVd inoculation, strong, moderate, and weak induction of *CEVI-1* (Solyc01g006300) occurs in tomato leaves, stems and, roots, respectively. In healthy plants, increased *CEVI-1* mRNA occurs after treatment of leaves with ethephon. Our microarray analysis showed *CEVI-1* regulation was variable and correlated with the infection stage, strain, and tissue/organ. In addition to M at 17 dpi, down-regulation was also observed at 49 dpi in S23-infected roots, but it was up-regulated at 24 dpi. Interestingly, our previous transcriptome analysis of PSTVd-infected leaves showed up-regulation by the M variant at 24 dpi and by S23 at all three time points. In line with the CEVd, the fold change of expression in leaves was at least twice as high than in roots. 

CEVd-infected cells reportedly exhibit altered cell wall composition and structure [67,68]. Consistent with this finding, our both root analysis, microarray and RNA-seq, revealed changes in the expression of many cell-wall related genes (Table S10). Repression of *Exp*, which encode expansins, and *Cel*, which encodes endo-1,4-beta-D-glucanases, occurred in both infections. However, more genes were regulated in S23 infection, a finding that is consistent with the observed root phenotype. Expansins are cell-wall-loosening proteins that are involved in lateral root formation, root hair growth, and resistance to biotic and abiotic stresses [69,70,71]. Lateral root formation inhibition occurs in *Arabidopsis* plants with *AtEXPA17* knockdown, but overexpression promotes the opposite effect [72]. After treatment with cyanamide, Soltys et al. [73] observed improper cell remodeling and tomato root growth inhibition elicited by lower expression of *LeEXP9* and *LeEXP18*, both expansins are responsible for proper root tip development [74,75]. We observed decreased *LeEXP18* (Solyc06g076220) and *LeEXP9* (Solyc06g005560) expression at 17 and 24 dpi in M-infected root and at 17 dpi in S23-infected roots. At 49 dpi, *LeEXP9*, but not *LeEXP18*, expression was elevated. The lower *Exp2* expression is reportedly correlates with inhibited cell growth and observed characteristic flat-top symptoms on the shoot of tomato “Rutgers” infected with PSTVdintU257A mutant [76]. Interestingly, in our previous study, *Exp2* expression in leaves was only altered (elevated) at 17 dpi in M infection, but in the present study, we observed strong down-regulation of this gene with S23 infection at 17 and 49 dpi and with M infection at 49 dpi. This data indicates its possible role in root growth. *Cel1* silencing disrupts and shortens the cell wall structure of *Arabidopsis* stems and roots [77]. Its down-regulation was only observed in S23-infected roots that is in line with observed phenotype.

The naturally occurring tomato mutant *dpy*, which is BR deficient, has dark-green rugose, downward curling leaves and is shorter than the wild type plant. Moreover, in the untreated *dpy* mutant, *LeBR*1 is reduced, but in the BR-treated mutant, *LeBR1* is significantly higher. The putative protein from *LeBR1* translation shows high sequence similarity with BRU1 encoded by the BR-regulated *XET* gene [78]. Its expression is increased in elongating soybean stems [79]. Our results are consistent with this observation. *BR1* transcript was reduced at 17 dpi (along with other BR biosynthesis pathway genes) but elevated at 49 dpi, when these genes were up-regulated a regrowth of stem in S23 infection was observed. 

AP2/ERF TFs play important roles in plant development and growth, fruit ripening, defense response, and metabolism. They can modulate ET, GA, CK, and ABA biosynthesis and are involved in response to AUX, CK, GB, and ABA [80]. For S23-infected roots, three genes that encode dehydration-responsive element binding protein (DREB) from AP2/ERF were down-regulated. Guo and Wang [81] observed *LeDREB2* (Solyc12g008350) expression in all analyzed organs (young and mature leaves, stem, and roots), with the highest level in root under normal conditions. This data indicates its role in plant growth and development. Overexpression in *Arabidopsis* of *CmERF053* from *Chrysanthemum morifolium*, which belongs to the DREB subfamily, significantly increases the lateral root growth/development compared to wild type [82]. Thus, the down-regulation of *DREB* genes in our experiments may be one of many factors related to the observed growth alterations in roots upon infection with the severe strain.

Lignification provides structural rigidity and durability to plant tissue. As reviewed by Miedes and coauthors [83] induction of lignin or lignin-like phenolic polymer synthesis and their deposition in the cell wall can be triggered in response to biotic and abiotic stresses. Lignin content in the secondary cell wall was higher in PSTVd-infected compared to control roots; however, the difference was only statistically significant for S23 infection (Figure 6). Contrary to our results, a marked decrease (70%) in lignin occurs in cultured chamamile cv. ‘Nowbona’ and tomato cv. ‘Heinz’ roots infected with PSTVd-C3 and PSTVd-AS1 compared to mock-inoculated plants [84]. Differences in the observation may be due to infection with different viroid variants, plant species, or cultivars used for infection. It should also be noted that lignin biosynthesis is a complex process; its components may be synthesized by different routes, and enzymes involved in its biosynthesis are usually derived from multigene families [85]. Complex crosstalk among lignin biosynthesis, growth, and defense occurs in *Arabidopsis*; the relationship of lignin content with growth and defense is difficult to predict [52]. A lignin content increase in response to pathogen attack is reported frequently. As described by Lauvergeat and associates [86], *AtCCR1* in *Arabidopsis* is preferentially expressed during development in tissues that are lignified, while *AtCCR2* expression is highly up-regulated in response to pathogenic bacteria *Xanthomonas campestris* infection. These data suggest its role in the biosynthesis of phenolics associated with hypersensitive response (HR). Exogenously applied allelochemicals increase production of lignin and its main monomers, changes that increase cell wall stiffness and inhibit root growth of soybean (*Glycine max* L. Merrill) [87]. The simultaneous silencing of both *CAD* and *CCR* reduces lignin content in tobacco [88] and *A. thaliana* [89]. However, only in *Arabidopsis* is this phenomenon associated with significantly altered plant development (such as dwarfism and sterility).

Intriguingly, we observed altered expression of photosynthesis-related genes. Roots are not considered photosynthetic organs, but plastid genes for photosynthetic proteins are constitutively transcribed in spinach root amyloplast [90]. Moreover, in *A. thaliana* [91] and *Camelina sativa* [92] roots, photosynthetic genes are differentially regulated under abiotic stresses. Chloroplasts may develop in *Arabidopsis* roots after shoot removal; this process is positively regulated by CK [93]. Study of transcriptomic profiles of camelina in response to salinity stress indicated that genes related to photosynthesis are down-regulated in shoots, while in roots, a gene that codes for chlorophyll-binding protein involved in photosystem II (PSII) is up-regulated 200-fold [92]. Our RNA-seq results correlate with this observation. In M-infected roots, two nuclear genes that encode chlorophyll a-b binding proteins were up-regulated and two others were down-regulated. Comparatively, in S23-infection, one was down-regulated and five were up-regulated. In both infections, two of these genes were very strongly activated. Similarly, more plastid genes related to PSI and II were regulated in S23-infection, and most of them were up-regulated, as well as those involved in the Calvin cycle. Differential regulation was also observed at 49 dpi, and more DEGs were suppressed in both infections. Many studies on viroid systemic leaf infection indicate strong down-regulation of chloroplast- and photosynthesis-related genes. For HSVd infection in cucumber, these changes are correlated with inhibition of photosynthesis [21]. The observed up-regulation in photosynthesis-related genes in roots could represent a response to reduced photosynthesis and may be involved in regulation of balance between growth and defense.

Taken together, our study provides a solid foundation of gene expression in tomato roots over the time-course of mild and severe PSTVd infection. Studying roots is essential to fully understand the viroid infection process and the plant defenses against it.

## Figures and Tables

**Table 1:** Enriched Kyoto Encyclopedia of Genes and Genomes (KEGG) pathway from microarray analysis.

Pathway	PSTVd-M			PSTVd-S23		
	17 dpi	24 dpi	49 dpi	17 dpi	24 dpi	49 dpi
**Down-regulated**						
Biosynthesis of secondary metabolites	2.50 × 10^−4^	4.06 × 10^−2^		2.03 × 10^−2^	2.25 × 10^−4^	4.66 × 10^−4^
Phenylpropanoid biosynthesis	4.27 × 10^−4^			4.60 × 10^−3^		
Plant hormone signal transduction		1.77 × 10^−2^			1.37 × 10^−3^	
Biosynthesis of unsaturated fatty acids		2.20 × 10^−2^				
Lysine degradation		2.20 × 10^−2^				
Carotenoid biosynthesis					2.31 × 10^−5^	
Regulation of autophagy						9.90 × 10^−3^
**Up-regulated**						
alpha-Linolenic acid metabolism	1.04 × 10^−2^	7.72 × 10^−7^	9.53 × 10^−3^	1.65 × 10^−3^		
Plant hormone signal transduction	1.04 × 10^−2^	1.19 × 10^−5^	3.08 × 10^−2^	1.22 × 10^−2^		
Biosynth of secondary metabolites		2.52 × 10^−4^	6.62 × 10^−7^	3.23 × 10^−2^		6.40 × 10^−5^
Endocytosis	2.26 × 10^−2^	1.01 × 10^−3^				
Ribosome			7.99 × 10^−13^			3.55 × 10^−12^
Steroid biosynthesis			1.14 × 10^−4^			8.13 × 10^−3^
Cysteine and methionine metabolism			1.60 × 10^−4^			1.24 × 10^−4^
Plant-pathogen interaction			2.53 × 10^−2^			4.99 × 10^−2^
Biosynthesis of amino acids			4.54 × 10^−3^			1.12 × 10^−6^
Protein processing in ER			9.19 × 10^−3^			1.56 × 10^−2^
Phenylalanine, tyrosine and tryptophan biosynthesis			3.10 × 10^−2^			9.77 × 10^−4^
Proteasome			3.16 × 10^−2^			7.98 × 10^−6^
Carbon metabolism						4.43 × 10^−5^
Carbon fixation in photosynthetic organisms				1.65 × 10^−3^		4.35 × 10^−2^
Carotenoid biosynthesis	2.26 × 10^−2^			3.23 × 10^−2^		
Glyoxylate and dicarboxylate metabolism			4.86 × 10^−2^			
Spliceosome						1.24 × 10^−4^
Peroxisome						3.88 × 10^−3^
Oxidative phosphorylation						8.13 × 10^−3^
Phagosome						2.49 × 10^−2^

Selected top categories are presented. q value is provided for each enriched pathway at indicated time.

## Supplementary Materials

The following are available online at https://www.mdpi.com/1999-4915/11/11/992/s1, Figure S1: Correlation of microarray and RNA-seq data for DEGs identified in microarray. Table S1: Probes used in the NanoString nCounter gene expression analysis. Table S2. Tomato DEGs identified in PSTVd-M- and PSTVd-S23-infected roots by microarray. Table S3. Summary of sequencing libraries. Table S4. List of DEGs identified from RNA-seq analysis after tomato infection with PSTVd M and S23 variants at 17 dpi. Table S5. KEGG pathway enrichment of DEGs from microarray analysis. Table S6. All enriched GO terms in the BP category for the down-and up-regulated DEGs (from microarray analysis) at the indicated infection time point. Table S7. All enriched GO terms in the BP category for the all down-and up-regulated DEGs (from RNA-seq analysis) by M and S23 variants, and for DEGs exclusively regulated by only one PSTVd variant. Table S8. Differentially regulated hormone-related genes identified by microarray and RNA-seq. Table S9. Differentially regulated genes encoding TFs and PKs identified by microarray and RNA-seq. Table S10. Differentially regulated cell-wall-related genes identified by microarray and RNA-seq. Table S11. Differentially regulated lignin-related genes identified by microarray and RNA-seq. Table S12. Differentially regulated proteasome-related genes.
