# Supplementary figures and images for: Root Transcriptomic Analysis Reveals Global Changes Induced by Systemic Infection of Solanum lycopersicum with Mild and Severe Variants of Potato Spindle Tuber Viroid
Source: Viruses. 2019 Oct 29;11(11):992. doi: 10.3390/v11110992 (PMC6893655; doi:10.3390/v11110992)

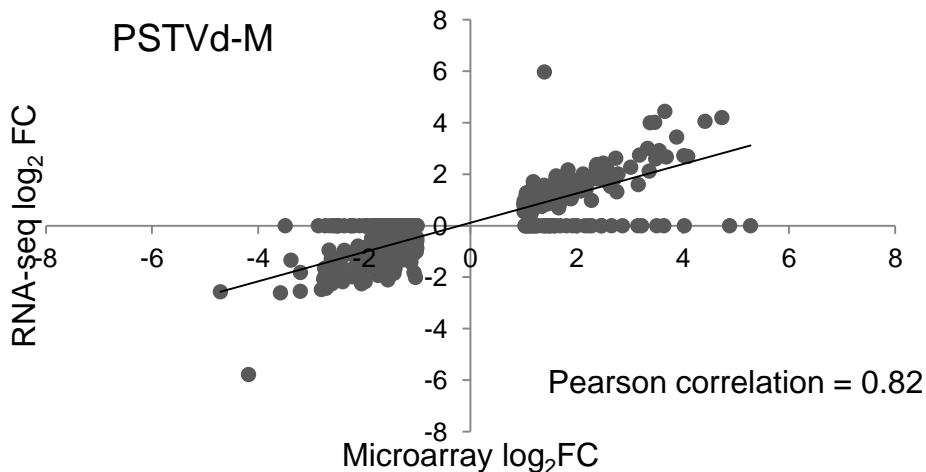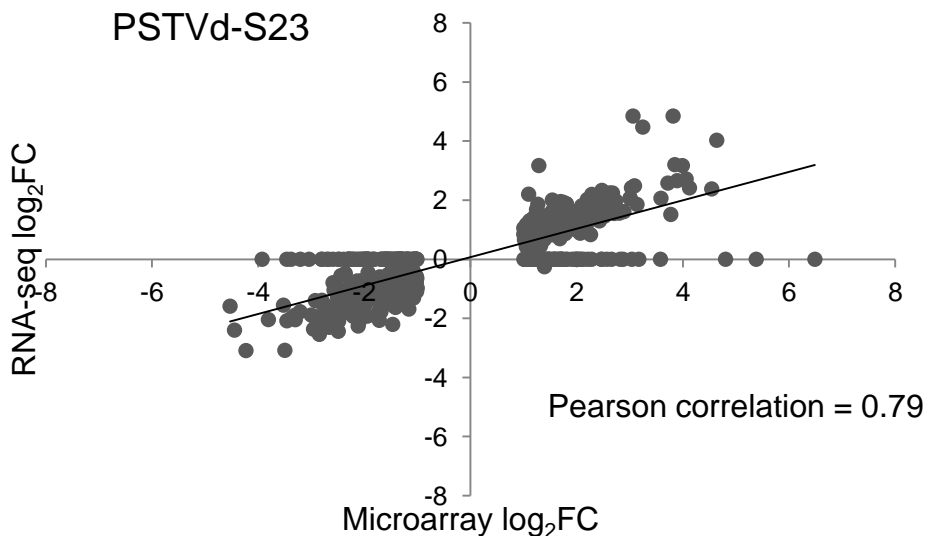

Supplement: Supplementary file 1 [file viruses-11-00992-s001.zip › viruses-619454-supplementary/Figure S1.pdf]
